# Intensive compared with less intensive blood pressure control to prevent adverse cardiac remodelling in children with chronic kidney disease (HOT-KID): a parallel-group, open-label, multicentre, randomised, controlled trial

**DOI:** 10.1016/S2352-4642(22)00302-9

**Published:** 2023-01

**Authors:** Manish D Sinha, Haotian Gu, Abdel Douiri, Janette Cansick, Eric Finlay, Rodney Gilbert, Larissa Kerecuk, Andrew Lunn, Heather Maxwell, Henry Morgan, Mohan Shenoy, Rukshana Shroff, Pushpa Subramaniam, Jane Tizard, Yincent Tse, Reza Rezavi, John M Simpson, Phil J Chowienczyk

**Affiliations:** a British Heart Foundation Centre, King's College London, London, UK; b Department of Medical Statistics, School of Population Health and Environmental Sciences, King's College London, London, UK; c Division of Imaging Sciences, King's College London, London, UK; d Department of Paediatric Nephrology, Evelina London Children's Hospital, Guy's and St Thomas' NHS Foundation Trust, London, UK; e Department of Paediatric Cardiology, Evelina London Children's Hospital, Guy's and St Thomas' NHS Foundation Trust, London, UK; f Department of Paediatrics, Medway Maritime Hospital, Medway, UK; g Department of Paediatric Nephrology, Leeds General Infirmary, Leeds, UK; h Department of Paediatric Nephrology, Southampton General Hospital, Southampton, UK; i Department of Paediatric Nephrology, Birmingham Children's Hospital, Birmingham, UK; j Department of Paediatric Nephrology, Nottingham University Hospital NHS Trust, Nottingham, UK; k Department of Paediatric Nephrology, Glasgow Royal Infirmary, Glasgow, UK; l Department of Paediatric Nephrology, Alder Hey Children's Hospital, Liverpool, UK; m Department of Paediatric Nephrology, Royal Manchester Children's Hospital, Manchester, UK; n Department of Paediatric Nephrology, UCL Great Ormond Street Hospital and Institute of Child Health, London, UK; o Department of Paediatrics, St Georges Hospital, London, UK; p Department of Paediatric Nephrology, Bristol Royal Hospital for Children, Bristol, UK; q Department of Paediatric Nephrology, Great North Children's Hospital, Newcastle upon Tyne, UK

## Abstract

**Background:**

Optimal target blood pressure to reduce adverse cardiac remodelling in children with chronic kidney disease is uncertain. We hypothesised that lower blood pressure would reduce adverse cardiac remodelling.

**Methods:**

HOT-KID, a parallel-group, open-label, multicentre, randomised, controlled trial, was done in 14 clinical centres across England and Scotland. We included children aged 2–15 years with stage 1–4 chronic kidney disease—ie, an estimated glomerular filtration rate (eGFR) higher than 15 mL/min per 1·73 m^2^—and who could be followed up for 2 years. Children on antihypertensive medication were eligible as long as it could be changed to angiotensin-converting enzyme (ACE) inhibitors or angiotensin receptor blockers (ARBs) if they were not already receiving these therapies. Participants were randomly assigned (1:1) to standard treatment (auscultatory office systolic blood pressure target between the 50th and 75th percentiles) or intensive treatment (systolic target <40th percentile) by the chief investigator using a rapid, secure, web-based randomisation system. ACE inhibitors or ARBs were used as first-line agents, with the dose titrated every 2–4 weeks to achieve the target blood pressure levels. The primary outcome was mean annual difference in left ventricular mass index (LVMI) by echocardiography measured by a masked observer and was assessed in the intention-to-treat population, defined as all the children who underwent randomisation irrespective of the blood pressure reached. Secondary and safety outcomes were the differences between groups in mean left ventricular relative wall thickness, renal function, and adverse effects and were also assessed in the intention-to-treat population. This trial is registered with ISRCTN, ISRCTN25006406.

**Findings:**

Between Oct 30, 2012, and Jan 5, 2017, 64 participants were randomly assigned to the intensive treatment group and 60 to the standard treatment group (median age of participants was 10·0 years [IQR 6·8–12·6], 69 [56%] were male and 107 [86%] were of white ethnicity). Median follow-up was 38·7 months (IQR 28·1–52·2). Blood pressure was lower in the intensive treatment group compared with standard treatment group (mean systolic pressure lower by 4 mm Hg, p=0·0012) but in both groups was close to the 50th percentile. The mean annual reduction in LVMI was similar for intensive and standard treatments (–1·9 g/m^2·7^ [95% CI –2·4 to –1·3] *vs* –1·2 g/m^2·7^ [–1·5 to 0·8], with a treatment effect of –0·7 g/m^2·7^ [95% CI –1·9 to 2·6] per year; p=0·76) and mean value in both groups at the end of follow-up within the normal range. At baseline, elevated relative wall thickness was more marked than increased LVMI and a reduction in relative wall thickness was greater for the intensive treatment group than for the standard treatment group (–0·010 [95% CI 0·015 to –0·006] *vs* –0·004 [–0·008 to 0·001], treatment effect –0·020 [95% CI –0·039 to –0·009] per year, p=0·0019). Six (5%) participants reached end-stage kidney disease (ie, an eGFR of <15 mL/min per 1·73 m^2^; three in each group) during the course of the study. The risk difference between treatment groups was 0·02 (95% CI −0·15 to 0·19, p=0·82) for overall adverse events and 0·07 (−0·05 to 0·19, p=0·25) for serious adverse events. Intensive treatment was not associated with worse renal outcomes or greater adverse effects than standard treatment.

**Interpretation:**

These results suggest that cardiac remodelling in children with chronic kidney disease is related to blood pressure control and that a target office systolic blood pressure at the 50th percentile is close to the optimal target for preventing increased left ventricular mass.

**Funding:**

British Heart Foundation.

## Introduction

Blood pressure is thought to be a key determinant of major clinical outcomes in children with chronic kidney disease, including progression of kidney disease and development of cardiovascular disease. The Effect of Strict Blood Pressure Control and ACE Inhibition on the Progression of Chronic Kidney Disease in Pediatric Patients (ESCAPE) trial showed that lowering ambulatory blood pressure targets slows kidney disease progression and remains the basis for current clinical recommendations.^1^, ^2^, ^3^ Guidelines for blood pressure targets differ somewhat between European and US societies and mainly reference the use of ambulatory blood pressure monitoring. Ambulatory blood pressure monitoring might be more closely related to target organ damage than office blood pressure, but its measurement is resource intensive and it is not always acceptable or practical for serial monitoring. Although the 2021 Kidney Disease Improving Global Outcomes guidelines recommend annual ambulatory blood pressure measurements to prevent adverse outcomes in children with chronic kidney disease, the need for data from randomised controlled trials using standardised office-based measurement of blood pressure to help define targets for treatment is also outlined in these guidelines.^4^

**Research in context**

**Evidence before this study**

Current recommendations regarding the management of blood pressure in children with chronic kidney disease are heavily based on data from a single trial, the Effect of Strict Blood Pressure Control and ACE Inhibition on the Progression of Chronic Kidney Disease in Pediatric Patients (ESCAPE) trial, with slower kidney disease progression in those with lower blood pressure targets (24 h ambulatory mean arterial pressure <50th percentile). However, recommended targets when using office-based blood pressure measurement are not based on as robust evidence and are higher (auscultatory systolic blood pressure target <90th percentile). Children with chronic kidney disease are predisposed to the development of left ventricular hypertrophy, which is associated with adverse clinical outcomes in early adulthood, but the optimal blood pressure to prevent adverse ventricular remodelling leading to left ventricular hypertrophy is uncertain. Effects of lower compared with higher blood pressure control were studied in a subset of children in the ESCAPE trial with no effect seen on left ventricular mass index (LVMI), but not all participants who underwent randomisation were studied.

**Added value of this study**

To our knowledge, HOT-KID is the first randomised controlled trial to examine the effect of different levels of blood pressure control on left ventricular remodelling in children with chronic kidney disease. On an intention-to-treat basis, the trial showed no significant effect of intensive office blood pressure reduction (target <40th percentile) compared with standard reduction (target 50th–75th percentile) on the primary outcome: LVMI. However, probably because of better-than-expected blood pressure control on entry to the study, left ventricular hypertrophy was present in only a small proportion of the participants, and LVMI was within the normal range. In contrast to LVMI, relative wall thickening, a measure of remodelling that might precede left ventricular hypertrophy and is itself related to adverse outcomes, was elevated at baseline and was significantly reduced by intensive compared with standard blood pressure reduction. This was despite the blood pressure in the standard treatment group being close to the 50th percentile.

**Implications of all the available evidence**

Although the HOT-KID trial has limitations, as it is a small, open-label trial, it supports the concept that a blood pressure target lower than the 50th percentile is close to the optimal for preventing progression of adverse ventricular remodelling. In combination with results of the ESCAPE study, a blood pressure threshold of lower than 50th percentile can be recommended as likely to prevent both progression of kidney disease and adverse ventricular remodelling. A remaining challenge is to clarify the best method of monitoring blood pressure in children and the equivalence of blood pressure thresholds.

Definitive evidence to determine the optimal blood pressure target in children to reduce adverse events requires follow-up in sufficiently large numbers over many years to obtain data on clinical events. Such an undertaking is not regarded as feasible currently. In children with chronic kidney disease, the strong association of cardiac remodelling—left ventricular hypertrophy in particular—with adverse cardiac events later in adulthood has led to a focus on cardiac remodelling as a surrogate outcome in this population.^5^, ^6^, ^7^, ^8^ A substudy of ESCAPE in 84 participants examined the effects of differing blood pressure targets on left ventricle remodelling and showed no significant effect, although the study did not include all patients who underwent randomisation and had variable follow-up between 12 months (n=28) and 24 months (n=56).^9^ Data on the relationship between cardiac remodelling and blood pressure have otherwise been observational and thus subject to confounding.^7^, ^8^, ^10^, ^11^, ^12^

Our previous work using auscultatory office blood pressure highlighted that left ventricular hypertrophy was closely associated with blood pressure and was less likely to occur in patients with blood pressure lower than the 50th percentile.^7^ As part of the Hypertension Optimal Treatment in Children with Chronic Kidney Disease (HOT-KID) study, we aimed to assess whether intensive treatment (systolic office blood pressure target <40th percentile) would be effective in reducing left ventricular mass and reverse maladaptive left ventricular remodelling in children with predialysis chronic kidney disease compared with standard treatment (systolic office blood pressure target between the 50th and 75th percentile). The 50th–75th percentile was regarded as standard treatment because, although some guidelines specify the 50th–95th percentile,^3^, ^4^ current practice in the light of the ESCAPE trial is to use a lower target.^1^, ^2^

## Methods

### Study design and participants

The HOT-KID study was a parallel-group, open-label, multicentre, randomised, controlled trial. We also completed an observational study as part of HOT-KID, but the results of this study will be reported at a later date. The randomised controlled trial was done at 14 clinical centres across England and Scotland. Children aged 2–15 years were included if they had stage 1–4 chronic kidney disease^13^—ie, an estimated glomerular filtration rate (eGFR) higher than 15 mL/min per 1·73 m^2^, with the eGFR calculated using the Schwartz formula^14^—in the preceding 12 months, and if it was anticipated that participants could be followed up for at least 2 years and attend all hospital clinical appointments and trial-related assessments in the UK. Children were eligible to enter the study while on antihypertensive therapy as long as, after inclusion in the trial, the class of their antihypertensive medication could be changed to angiotensin-converting enzyme (ACE) inhibitors or angiotensin receptor blockers (ARBs; if they were not already on these drugs) and if antihypertensive drugs could be withdrawn or added or doses could be modified to achieve the allocated target blood pressure level. Use of ACE inhibitors or ARBs was specified so that results of the trial would be representative of children treated with a regimen based on these drugs, which is regarded as standard care. Individuals were excluded if they had an arteriovenous fistula, were on dialysis at the time of study entry, or had a previous kidney transplant. A full list of inclusion and exclusion criteria and flowchart is provided in the appendix (pp 3–4).

The trial design complies with the principles of the Declaration of Helsinki and was approved by the Clinical Studies Group of the British Association for Paediatric Nephrology, with recruitment and follow-up supported by all paediatric nephrology centres in the UK. Parents or legal guardians of children gave written informed consent, and participants gave assent as appropriate. Trial participants were enrolled by the study site principal investigator with any queries regarding eligibility discussed with chief investigator (MDS). The study protocol (incorporating the randomised controlled trial) had no additional amendments to that included in the appendix (pp 19–39) and was approved by the UK National Research Ethics Committee (10/H0802/13), participating institutions, and relevant regulatory authorities.

### Randomisation and masking

Participants were randomly assigned (1:1) to receive standard treatment or intensive treatment by the chief investigator (MDS) using a rapid, secure, web-based randomisation system developed in house by the King's Clinical Trials Unit (King's College London). Randomisation was stratified according to clinical centre. Participants and recruiting clinicians were informed of the participant's allocation and trial-related systolic blood pressure targets electronically. Therefore, no unblinding or code breaking was required. Clinicians performing investigations were masked to participants' systolic blood pressure percentile and target, and the trial team and recruiters were masked to results.

### Procedures

On the day of baseline assessments (day 0), eligible participants, following consent, underwent standardised measurement of blood pressure, echocardiography, and other study-related investigations, including measurement of plasma creatinine. Assessments were performed in dedicated clinical research facilities where possible, with participants aware of planned trial-related assessments.

After randomisation, antihypertensive treatment in children whose blood pressure was not at the target level was commenced or modified to achieve the allocated target. For the intensive treatment group, the systolic office blood pressure target was lower than the 40th percentile (Z score –0·26), and for the standard treatment group it was between the 50th and 75th percentile (Z score 0·00–0·67). ACE inhibitors or ARBs were used as first-line agents, with the dose titrated every 2–4 weeks to achieve the target blood pressure levels. In cases in which children were on drugs other than ACE inhibitors or ARBs, an ACE inhibitor or ARB was substituted for another class of drug (with no washout period). Following ACE inhibitors or ARBs, the following drugs were used based on physicians' preferences to achieve target blood pressure: calcium channel blockers, β-receptor blockers, diuretics, or **α-**channel blockers. The use of long-acting drugs with once daily dosing was recommended. Following achievement of the target blood pressure, all children were monitored at least every 4 months at the time of routine hospital appointments, and their office blood pressure was maintained in the assigned target range over the duration of the study. Up-titration, down-titration, or withdrawal of antihypertensive drugs during the trial was determined using the flowcharts shown in the appendix (pp 6–7). Trial-related blood pressure targets for all participants were provided at baseline and at annual intervals for the duration of participation of all children.^15^

Data were collected by means of a standardised data collection form, with subsequent data entry into a dedicated web-based database (MedSciNet). At each follow-up visit, height, weight, blood pressure, antihypertensive medication, antihypertensive drug adherence, concomitant medications, and adverse outcomes were recorded. Laboratory data (haemoglobin, urea, creatinine, electrolytes, albumin, calcium, phosphate, intact parathyroid hormone, and proteinuria) were obtained at baseline and every year thereafter, with additional renal function assessments after an increase in dose of ACE inhibitors or ARBs, or as deemed necessary by the clinician.

Office blood pressure was measured by a trained staff member (physician or nurse) and was measured by the same person on each child where possible. Patients were required to rest for at least 5 min in a seated position, systolic blood pressure was initially confirmed by palpation, and then the blood pressure was measured three times in quick succession using auscultation and aneroid sphygmomanometer with an appropriately sized cuff by inflating the cuff higher than systolic blood pressure. Cuff size was selected by ensuring cuff bladder length and width according to standard guidelines.^2^, ^3^ The same validated blood pressure instrument (Welch Allyn Dura-Shock DS54 or WelchAllyn DuraShock DS-66 trigger model, Welch Allyn New York, NY, USA) and range of cuff sizes were provided to all participating centres. The same protocol was used for monitoring blood pressure between annual visits (usually at intervals of no more than 3 months at the local physician's discretion), with titration of drugs performed according to the flowcharts in the appendix (pp 6–7) at these intermediate visits if blood pressure was outside the target. Ambulatory blood pressure monitoring was not required for participation in the trial but was performed by clinicians if this was the practice at their centre.

Transthoracic echocardiograms were acquired at baseline and at annual intervals by a core team of experienced echocardiographers according to a standardised research protocol. To reduce inter-observer error, investigators travelled to all external centres from the lead centre (Evelina London Children's Hospital, King's College London) and performed all study-related cardiovascular assessments. Each echocardiogram was transferred in DICOM format and stored on a database in the Clinical Research Facilities, St Thomas' Hospital, London, UK, where a single masked investigator (HG) performed the overall image analysis for the primary outcome without knowledge of allocation to treatment group. The intra-observer coefficient of variation for this investigator was 8·5% for left ventricular mass and 7·1% for relative wall thickness. Left ventricular mass varies widely across the paediatric age range (0−16 years). Therefore, to allow standardisation, it is expressed as left ventricular mass index (LVMI; left ventricular mass in grams divided by height in metres raised to the allometric power of 2·7 [g/m^2·7^]) as a measure that accounts for body size.^16^, ^17^ Left ventricular hypertrophy was defined as a LVMI greater than or equal to the 95th percentile using age-specific reference intervals for children without chronic kidney disease.^18^ To adjust for somatic growth and to allow for more precise measurement of change in left ventricular mass, we also calculated left ventricular mass for height Z scores.^19^ Relative wall thickness, defined as two times posterior wall thickness divided by the left ventricular diastolic diameter, was measured to assess left ventricular geometry.^16^ Strict quality control processes based on American Society of Echocardiography recommendations were applied (appendix p 8).^16^

### Outcomes

The primary outcome measure was the difference in LVMI (expressed in g/m^2·7^) between the intensive versus standard treatment groups. To take into account the variable follow-up period, the mean annual change in LVMI was calculated. We also report changes in mean left ventricular mass for height Z score. As secondary outcomes, to assess the effect on left ventricular reverse remodelling, we analysed the difference in mean relative wall thickness between the trial groups. We report systolic and diastolic blood pressure in both groups and the proportion of participants requiring antihypertensive medications (together with number of antihypertensive medications). Safety and renal (eGFR) outcomes were additional reported secondary outcomes. Adverse events were monitored by study investigators and logged as appropriate. Potential adverse effects of the drugs were well known to the investigators and were managed according to standard care and according to the protocol. Serious side-effects were reported by investigators on adverse event forms (appendix p 36) and reviewed by investigators (MDS and PJC). Reasons for study withdrawals, treatment discontinuation, possible side-effects, and renal outcomes were also documented by study investigators.

### Statistical analysis

On the basis of existing information on LVMI and change in LVMI at annual intervals at proposed blood pressure levels in children with chronic kidney disease, the original sample size was set to detect a difference in change in mean LVMI of more than 3·1 g/m^2·7^ over a 2-year follow-up period between the intensive versus the standard treatment groups, assuming a LVMI SD of 6·0 g/m^2·7^. This difference was considered clinically significant because it is a third of the difference (9·4 g/m^2·7^) between participants previously studied in our cohort of children with chronic kidney disease who had blood pressure in the 50th–75th percentile or lower than the 40th percentile. We estimated that 144 participants were required over the 2-year duration to detect this difference with 90% power (significance p<0·05), assuming a dropout rate of up to 20%. A planned blinded interim analysis of the variation in LVMI, after 1 year of follow-up in all participants in the study (not by randomisation) confirmed that this was similar (6·7 *vs* 6·0 g/m^2·7^) to that used in the original sample size calculation. Recruitment was slower than expected, and trial recruitment was terminated due to funding constraints at 124 participants. However, at an interim review, it was apparent that the reduced sample size would be mitigated by a longer follow-up period, with the median follow-up being approximately 50% greater than originally anticipated. A formal sample size recalculation was not performed. The primary analysis was then changed to the change in mean LVMI over the duration of the trial, including all available data.

The primary analysis was the comparison of the outcome measures, assessed according to allocation on an intention-to-treat basis, defined as all the children who underwent randomisation irrespective of the blood pressure reached. No allowance was made for multiple testing of outcomes other than the primary outcome. We analysed the mean annual rate of change in LVMI (95% CI), and the difference (95% CI) in these rates using a restricted maximum likelihood-based repeated measures approach in combination with the Newton-Raphson algorithm. The analysis included the fixed, categorical effects of treatment, clinical centre, visit, and treatment-by-visit interaction, as well as the continuous, fixed covariates of baseline value of the outcome measure and baseline value-by-visit interaction. We used a common unstructured covariance structure to model the within-patient errors. The Kenward-Roger approximation was used to estimate denominator degrees of freedom. Significance tests were based on least-squares means using a two-sided α value of 0·05 (two-sided 95% CIs).

This model accounts for the baseline and all subsequent follow-up measurements, and enables the inclusion of participants with missing measurements. It assumes that the outcome measure changes linearly over time. Patients were followed up for between 1 year and 5 years from baseline and, like all linear mixed models, the model assumes that when data were missing, they were missing at random. We also did a post-hoc sensitivity analysis that relaxes the assumption of a linear change of outcome variable over time was as follows: we estimated mean LVMI (or other outcome measures), with 95% CIs, in each treatment group at each timepoint (from 1 year to 5 years), along with the absolute difference in these means (95% CIs), using a linear mixed-effects model for repeated measures. Subgroup analysis for the primary outcome by age (dichotomised according to the median) and sex was additionally performed post hoc.

We did a further post-hoc sensitivity analysis to evaluate the possible influence of missing values for the primary and secondary outcomes. Multiple imputation was done using Markov chain Monte Carlo with the following variables used to impute the missing values: clinical centre, group, time, and baseline measurements (height, weight, BMI, body surface area, heart rate, systolic blood pressure, diastolic blood pressure, mean arterial pressure, and pulse pressure). 20 datasets were imputed to generate the average adjusted mean difference reported previously. Adverse events and serious adverse events are summarised by treatment group in the intention-to-treat population. A Wilson test was used to assess difference in risk for overall adverse events and serious adverse events in the intention-to-treat population. The statistical analysis plan is in the appendix (pp 40–47). All statistical analysis was done in Stata (version 17). The study protocol was reviewed by the UK Medicines and Healthcare products Regulatory Agency and judged not to be a Clinical Trial of Investigational Medical Product study because it was open label and used established drugs; therefore, the need for independent safety monitoring was removed.

This trial is registered with ISRCTN, ISRCTN25006406.

### Role of the funding source

The funder of the study had no role in study design, data collection, data analysis, data interpretation, or writing of the report other than through providing comments by external reviewers.

## Results

**Figure 1:**
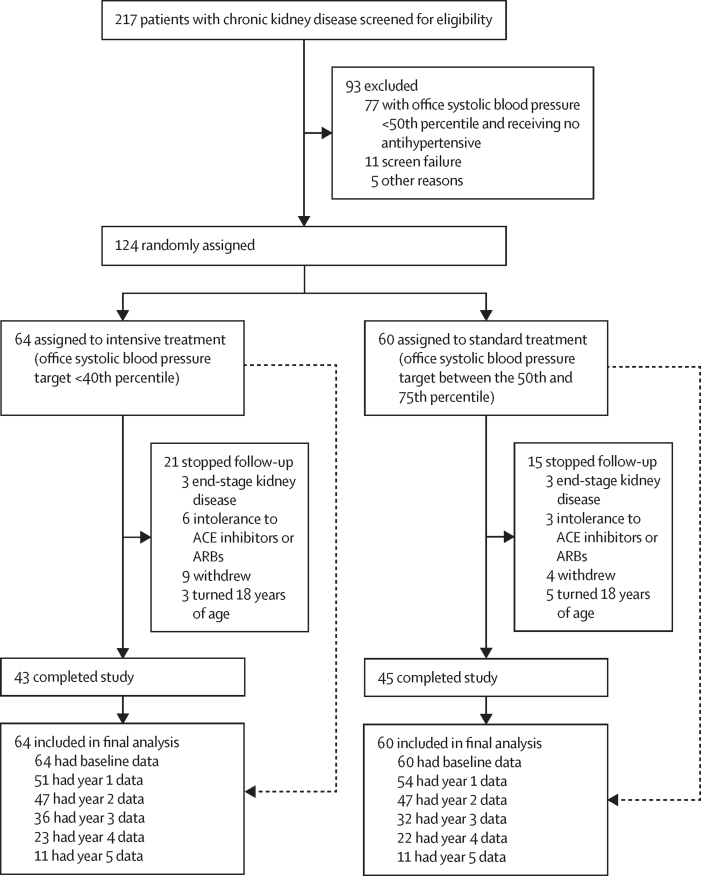
Trial profile At the final follow-up visit, the majority of patients had not reached 5 years of study participation, which accounts for the sharp decrease in numbers available for follow-up between year 4 and 5. ACE=angiotensin-converting enzyme. ARB=angiotensin receptor blocker.

Between Oct 30, 2012, and Jan 5, 2017, 64 participants were randomly assigned to the intensive treatment group and 60 to the standard treatment group, and were included in the intention-to-treat population (figure 1). Participants were followed up for a median of 38·7 months (IQR 28·1–52·2), with the final study visit on March 5, 2019. Baseline characteristics of the intention-to-treat population are shown in table 1. The median age of all participants was 10·0 years (6·8–12·6), 69 (56%) of 124 participants were male, and 107 (86%) were of white ethnicity. At baseline, six (9%) of 64 participants in the intensive treatment group and eight (13%) of 60 participants in the standard treatment group had an eGFR of less than 45 mL/min per 1·73 m^2^. The primary cause of chronic kidney disease in 39 (61%) participants in the intensive treatment group and 42 (70%) participants in the standard treatment group was congenital anomalies of the kidney and urinary tract, and chronic kidney disease was thus lifelong in these participants. Anaemia was treated with oral iron supplementation, and no trial participants received erythropoietin or immunosuppresants for glomerulopathies or other indications during the course of the trial. At baseline, mean clinic blood pressure was 107/63 mm Hg (SD 11/12), with a mean clinic blood pressure Z score of 0·55/0·06 (0·91/1·25); 38 (63%) participants' systolic blood pressure was lower than the 75th percentile in the standard treatment group, and in 12 (19%) participants in the intensive treatment group it was lower than the 40th percentile of the reference population. 80 (65%) participants were on antihypertensive medications at baseline, 52 (65%) of whom were on ACE inhibitors or ARBs alone, nine (11%) on a calcium channel blocker, three (4%) on other antihypertensives (all as monotherapy), and 16 (20%) on two or more antihypertensive medications including ACE inhibitors or ARBs. Blood pressure and echocardiographic measures at baseline between those on antihypertensives and those without antihypertensive medication were similar (appendix p 14).

**Table 1:** Baseline characteristics of the intention-to-treat population

		**Intensive treatment group (n=64)**	**Standard treatment group (n=60)**
Age, years		9·4 (6·2–12·4)	10·3 (7·3–12·8)
Sex			
	Male	34 (53%)	35 (58%)
	Female	30 (47%)	25 (42%)
Ethnicity			
	Asian	6 (9%)	6 (10%)
	Black	0	2 (3%)
	White	57 (89%)	50 (83%)
	Other	1 (2%)	2 (3%)
Primary renal disease			
	Congenital anomaly of the kidney and urinary tract	39 (61%)	42 (70%)
	Glomerulopathies	5 (8%)	7 (12%)
	Other	20 (31%)	11 (18%)
Height, cm		134·0 (114·7–151·5)	137·7 (124·6–150·5)
Weight, kg		30·9 (21·1–42·4)	32·9 (25·4–94·3)
BMI, kg/m^2^		17·0 (15·9–19·1)	18·0 (16·0–22·0)
Height, SD score		−0·22 (1·11)	−0·26 (1·27)
Weight, SD score		0·16 (1·30)	0·40 (1·27)
BMI, SD score		0·37 (1·24)	0·69 (1·26)
Body surface area, m^2^		1·07 (0·82–1·35)	1·11 (0·93–1·43)
Heart rate, bpm		81 (13·1)	82 (14·5)
Systolic blood pressure, mm Hg		107 (11)	108 (11)
Diastolic blood pressure, mm Hg		62 (11)	64 (13)
Systolic blood pressure, Z score		0·54 (0·98)	0·57 (0·84)
Diastolic blood pressure, Z score		0·02 (1·23)	0·10 (1·29)
Mean arterial pressure, mm Hg		77 (10)	78 (11)
Pulse pressure, mm Hg		45 (11)	45 (11)
Antihypertensive medications		43 (67%)	37 (62%)
	Angiotensin-converting enzyme inhibitor or angiotensin receptor blocker	30 (47%)	22 (37%)
	Calcium channel blocker	3 (5%)	6 (10%)
	Other	2 (3%)	1 (2%)
	Two or more medications	8 (1%)	8 (1%)
Biochemistry			
	Estimated glomerular filtration rate, mL/min per 1·73 m^2^	73·6 (57·3–94·4)	83·5 (59·6–98·8)
	Estimated glomerular filtration rate <45 mL/min per 1·73 m^2^	6 (9%)	8 (13%)
	Urinary albumin-to-creatinine ratio*, mg/mmol	9·6 (2·1–19·5)	1·60 (0·55–3·81)
	Haemoglobin, g/L	124·0 (111·0–130·0)	130·0 (121·0–140·0)
	Albumin, g/L	46·0 (42·0–47·0)	43·0 (41·0–48·0)
	Serum-corrected calcium, mmol/L	2·47 (2·37–2·56)	2·39 (2·33–2·44)
	Serum phosphate, mmol/L	1·40 (1·23–1·60)	1·40 (1·30–1·54)
	Serum intact parathyroid hormone, ng/L	27·0 (6·65–63·0)	29·0 (9·68–40·5)
	25-hydroxy vitamin D3, μg/L	60·0 (44·0–88·0)	65·2 (41·0–86·0)

Data are median (IQR), n (%), or mean (SD). Percentages might not sum to 100 as a result of rounding.

* Assessed in 21 patients in the intensive treatment group and 25 patients in the standard therapy group.

Throughout follow-up, the mean office systolic blood pressure was 103 mm Hg (SD 10) in the intensive treatment group versus 107 mm Hg (10) in the standard treatment group (p=0·0012), mean office diastolic blood pressure was 60 mm Hg (10) versus 64 mm Hg (12; p=0·0003), and mean arterial pressure was 75 mm Hg (9) versus 78 mm Hg (10; p=0·0001), with a mean systolic blood pressure Z score of 0·06 (0·88) versus 0·19 (0·80) and a mean diastolic blood pressure Z score of –0·27 (1·09) versus 0·01 (1·16). Over the course of the study, the number of antihypertensive medications prescribed per patient was higher in the intensive treatment group than in the standard treatment group, with a mean difference between groups of 0·50 (95% CI 0·25 to 0·75, p<0·0001; appendix p 9). To achieve the target blood pressure (appendix p 10), there was a sustained increase in the number of children on antihypertensive medications, those requiring an increase in dose, or those who required two or more antihypertensive medications; all of these increases were higher in the intensive treatment group than in the standard treatment group (appendix p 11). There was a decrease in heart rate in all patients over the course of the study that did not differ between the intensive and standard treatment groups (–5·4 bpm [13·1] *vs* –4·1 bpm [15·0]; p=0·65).

Participants were matched for most echocardiographic measures at baseline (appendix p 15). The mean LVMI for all participants was 31·1 g/m^2·7^ (SD 9·2), with relatively few children reaching the definition of left ventricular hypertrophy (four [6%] of 64 participants in the intensive treatment group *vs* five [8%] of 60 in the standard treatment group at baseline; appendix p 15), all with eccentric left ventricular hypertrophy. Mean relative wall thickness was higher than the 80th percentile of values previously reported in normal children,^20^ with values that were similar in intensive and standard treatment groups at baseline (0·34 [SD 0·05] *vs* 0·36 [0·06]; appendix p 15).

**Figure 2:**
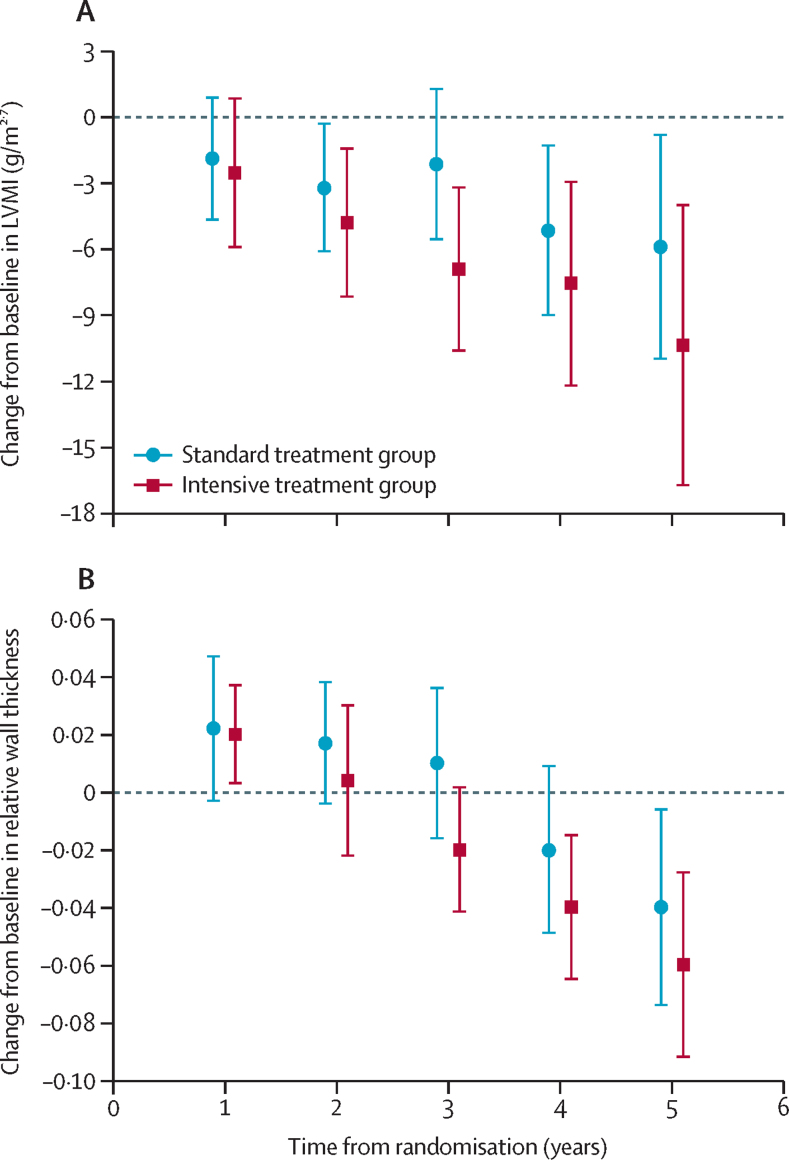
LVMI and relative wall thickness over time (A) Change in mean LVMI from baseline. Data are shown as mean, with error bars indicating 95% CIs. (B) Change in mean relative wall thickness, defined as from baseline. Data are shown as mean, with error bars indicating 95% CIs. Means were estimated by use of a linear mixed-effects model for repeated measures. LVMI=left ventricular mass index.

There was a sustained reduction in LVMI over the course of the trial, but the difference between groups did not reach statistical significance (figure 2A; table 2). The annual rate of change in left ventricular mass index was –1·9 g/m^2·7^ (95% CI –2·4 to –1·3) for the intensive treatment group versus –1·2 g/m^2·7^ (–1·5 to 0·8) for the standard treatment group (difference in means –0·7 g/m^2·7^ [95% CI –1·9 to 2·6] per year; p=0·76). Mean left ventricular mass for height Z score showed an annual change of –0·3 (–0·4 to –0·2) for the intensive treatment group versus –0·2 (–0·3 to –0·1) for the standard treatment group (difference in means –0·1 [95% CI –0·5 to –0·3] per year; p=0·68; appendix p 12). Multiple imputation analysis used to examine the potential influence of missing data on LVMI showed effect sizes and 95% CIs that were similar to those in the primary analysis (mean difference in annual progression of LVMI –0·7 g/m^2·7^ [95% CI –2·0 to 0·7] per year; p=0·30; data not shown in full).

**Table 2:** Left ventricular mass over time in the intention-to-treat population

	**Intensive treatment group**		**Standard treatment group**		**Difference between groups (95% CI)**	p value*
	n	Mean LVMI (95% CI)†, g/m^2·7^	n	Mean LVMI (95% CI)†, g/m^2·7^		
Change in mean (95% CI) LVMI per year, g/m^2·7^	..	−1·9 (−2·4 to −1·3)	..	−1·2 (−1·5 to 0·8)	−0·7 (−1·9 to 2·6)	0·76
Baseline	64	31·9 (29·4 to 34·4)	60	30·3 (28·2 to 32·4)	−1·6 (−4·9 to 1·6)	..
Year 1	51	29·4 (27·3 to 31·5)	54	28·4 (26·6 to 30·2)	−1·0 (−3·7 to 1·7)	0·47
Year 2	47	27·2 (25·2 to 29·1)	47	27·1 (25·2 to 29·0)	−0·1 (−2·7 to 2·6)	0·97
Year 3	36	25·1 (23·0 to 27·2)	32	28·2 (25·6 to 30·7)	3·1 (−0·2 to 6·3)	0·055
Year 4	23	24·4 (21·4 to 27·4)	22	25·2 (22·4 to 27·9)	0·8 (−3·2 to 4·7)	0·70
Year 5	11	21·6 (17·7 to 25·5)	11	24·4 (21·3 to 27·6)	2·8 (−1·9 to 7·5)	0·23

At the final follow-up visit, the majority of patients had not reached 5 years of study participation, which accounts for the sharp decrease in numbers available for follow-up between year 4 and 5. LVMI=left ventricular mass index.

* The p value denotes difference between the two groups in change in mean LVMI per year.

† Unless otherwise stated.

The two treatment strategies resulted in a reduction in relative wall thickness over time (figure 2B; table 3). The annual rate of change in relative wall thickness was greater in the intensive treatment group than in the standard treatment group (–0·010 [95% CI 0·015 to –0·006] *vs* –0·004 [–0·008 to 0·001]; difference in means –0·020 [95% CI –0·039 to –0·009] per year, p=0·0019). The left ventricular remodelling resulted in more children with normal geometry in the intensive treatment group than in the standard treatment group at all timepoints (appendix p 13). As with LVMI, multiple imputation analysis yielded similar results for relative wall thickness (difference in means –0·020 [95% CI –0·039 to –0·009] per year, p=0·0019; data not shown in full). Subgroup analysis for the primary outcome by age (dichotomised according to the median) and sex showed no difference between groups (appendix p 18).

**Table 3:** Relative wall thickness over time in the intention-to-treat population

	**Intensive treatment group**		**Standard treatment group**		**Difference (95% CI)**	p value*
	n	Mean relative wall thickness (95% CI)†	n	Mean relative wall thickness (95% CI)†		
Change in mean (95% CI) relative wall thickness per year	..	−0·010 (0·015 to −0·006)	..	−0·004 (−0·008 to 0·001)	−0·020 (−0·039 to −0·009)	0·0019
Baseline	64	0·34 (0·33 to 0·36)	60	0·36 (0·34 to 0·37)	0·013 (−0·007 to 0·034)	..
Year 1	51	0·36 (0·34 to 0·37)	54	0·38 (0·36 to 0·40)	0·019 (−0·004 to 0·043)	0·10
Year 2	47	0·35 (0·33 to 0·37)	47	0·37 (0·36 to 0·39)	0·026 (0·001 to 0·051)	0·042
Year 3	36	0·32 (0·30 to 0·34)	32	0·37 (0·35 to 0·38)	0·045 (0·020 to 0·070)	0·0005
Year 4	23	0·30 (0·28 to 0·32)	22	0·33 (0·30 to 0·36)	0·032 (−0·002 to 0·067)	0·068
Year 5	11	0·28 (0·25 to 0·31)	11	0·31 (0·28 to 0·35)	0·033 (−0·009 to 0·075)	0·12

Relative wall thickness is defined as two times posterior wall thickness divided by the left ventricular diastolic diameter. At the final follow-up visit, the majority of patients had not reached 5 years of study participation, which accounts for the sharp decrease in numbers available for follow-up between year 4 and 5.

* The p value denotes difference between the two groups in change in the mean relative wall thickness per year.

† Unless otherwise stated.

133 adverse events occurred over the course of the trial of which 15 were serious adverse advents (table 4). The difference in risk of adverse events between the intensive and standard treatment groups did not differ significantly: risk difference 0·02 (95% CI −0·16 to 0·19), p=0·83, for overall adverse events and 0·07 (−0·05 to 0·19), p=0·25, for serious adverse events. 28 (23%) participants were withdrawn or elected to withdraw over the course of the trial (appendix p 16). Reasons for withdrawal included not reaching the blood pressure target, intolerance to ACE inhibitors or ARBs, and progression to end-stage kidney disease (figure 1). Two (2%) children were unable to reach the blood pressure target (both in the intensive treatment group). Seven (6%; five [8%] in the intensive treatment group and two [3%] in the standard treatment group) children were unable to tolerate ACE inhibitors or ARBs because of an increase in plasma creatinine deemed to be clinically significant by their physician or symptoms of leg pains (one in the intensive treatment group) and dizziness (one in the standard treatment group; figure 1; table 4; appendix p 16). 11 (9%; seven [11%] in the intensive treatment group and four [6%] in the standard treatment group) participants requested to withdraw from the study for various reasons (appendix p 16). Six (5%) participants reached end-stage kidney disease (ie, they had an eGFR of <15 mL/min per 1·73 m^2^; three in each group) during the course of the study (figure 1; appendix p 16).

**Table 4:** Safety and renal outcomes related to the blood pressure in the intention-to-treat population

		**Intensive treatment group (n=64)**	**Standard treatment group (n=60)**
**Safety outcomes**			
Adverse events		77	56
Serious adverse events		9	6
Type of serious adverse events*			
	Need for renal-replacement therapy	3	3
	Decreased glomerular filtration rate	5	2
	Dizzy	0	1
	Leg pains	1	0
Anticipated adverse event			
	Decreased glomerular filtration rate	5	7
	Hyperkalaemia	2	2
	Cough	2†	2
	Dizziness	3‡	0
	Lethargy	3‡	2
	Headaches	3‡	2
	Gastrointestinal symptoms	12	11‡
Study-emergent adverse event			
	Allergic reaction to eggs	1	0
	Balanitis	3§	0
	Dental pain	0	1
	Otitis externa	0	1
	Eczema	1	0
	Nocturnal enuresis	1	0
	Epistaxis	2	0
	Eye swelling	1	0
	Fracture due to accident	3	2
	Gastrostomy leaking	1	0
	Gum hypertrophy	1	0
	Planned hospitalisations	1	3‡
	Unplanned hospitalisations	10	3
	Hot flushes	1	1
	Henoch-Schönlein purpura nephritis	0	2‡
	Knee pain and swelling	1	0
	Respiratory infections	7¶	6
	Mood disturbance	1	0
	Pyuria	4‡	5‡
	Urinary tract infection	5‡	2
	Urosepsis	1	0
	Transient leg swelling	0	1
	Transient transaminitis	1	0
	Rash	1	1
	Scabies	0	1
	Vitiligo	0	1
**Renal outcomes**			
>30% reduction in participants with eGFR <60 mL/min per 1·73 m^2^ at baseline		2/19 (11%)	3/15 (20%)
eGFR <45 mL/min per 1·73 m^2^ during the study		4/58 (7%)	2/52 (4%)
eGFR <15 mL/min per 1·73 m^2^ during the study		3 (5%)	3 (5%)

Data are n (%) or n/N (%). eGFR=estimated glomerular filtration rate.

* Resulted in withdrawal from the study.

† Changed from an angiotensin-converting enzyme inhibitor to angiotensinogen receptor blocker.

‡ Two episodes in the same child.

§ Same child (who eventually underwent circumcision).

¶ Three episodes in same child.

Over the course of the study, the mean difference in eGFR between the intensive treatment group and the standard treatment group was –3·9 mL/min per 1·73 m^2^ (95% CI –13·6 to 5·9, p=0·44). In participants with an eGFR of less than 60 mL/min per 1·73 m^2^ at baseline, two (11%) of 19 in the intensive treatment group and three (20%) of 15 participants in the standard treatment group had a greater than 30% reduction in eGFR over the course of the study (table 4). In participants with an eGFR greater than 45 mL/min per 1·73 m^2^ at baseline, the eGFR decreased below 45 mL/min per 1·73 m^2^ over the course of the study for four (7%) of 58 participants in the intensive treatment group and two (4%) of 52 participants in the standard treatment group (table 4).

## Discussion

To our knowledge, the HOT-KID study provides the first evidence from a randomised controlled trial on the effects of differing blood pressure targets on cardiac remodelling. The main findings of the HOT-KID study are that, in an intention-to-treat analysis, allocation to an office systolic blood pressure target lower than the 40th percentile versus a target higher than the 50th percentile did not result in a significant reduction in LVMI over the course of several years and that the 95% CIs for change in mean LVMI excluded the effect size we specified as clinically significant: a change of greater than 3·1 g/m^2·7^ over a 2-year follow-up period. The two treatment strategies resulted in a reduction in relative wall thickness over time. This contrasts with the increase that is seen in children in the general population.^20^ However, there was a greater reduction in concentric remodelling, as measured by relative wall thickness, in the intensive treatment group than in the standard treatment group. These findings suggest that the remodelling of the left ventricle is dependent on blood pressure target even over a narrow blood pressure range. The mean blood pressure of the study population at baseline was close to the 50th percentile. The 2021 Kidney Disease Improving Global Outcomes clinical practice guideline for management of blood pressure in children with chronic kidney disease recommends an office auscultatory systolic blood pressure target lower than the 90th percentile and 24 h mean arterial pressure measured by ambulatory blood pressure monitoring lower than the 50th percentile, but there is no clear rationale for the difference in target between the two different measures of blood pressure.^4^ A tendency to adopt a stricter target close to the 50th percentile when using office blood pressure might be driven by the findings of the ESCAPE study.^1^ In our study, because of the well controlled blood pressure at baseline, there was a low prevalence of left ventricular hypertrophy, with the mean LVMI below the 50th percentile of a reference population of healthy children.

In the context of a near-normal left ventricular mass in the majority of participants and a low prevalence of left ventricular hypertrophy, a change in LVMI is unlikely. In contrast to LVMI, relative wall thickness was elevated in the majority of children, reflecting a tendency to concentric remodelling. Concentric remodelling is a recognised feature of hypertension-mediated organ damage in clinical practice guidelines^2^, ^3^ and in adults there is evidence that it might be a more sensitive measure of target organ damage than LVMI.^21^ Therefore, the finding of a greater reduction in relative wall thickness with the lower blood pressure target is of relevance when considering the prevention of concentric remodelling.

Some children in the intensive treatment group did not reach a blood pressure lower than the 40th percentile, and the majority of children in the standard treatment group had blood pressure that was close to the 50th percentile. As a result, the blood pressure difference between the two groups was modest. This finding might be explained by the tendency of physicians, patients, and their families to be conservative when considering up-titrating or down-titrating therapy, leading to convergence of achieved blood pressure in the two groups. This convergence of achieved blood pressure is in keeping with trials of similar design. In the ESCAPE study, for example, target blood pressure in the two groups of the trial was 24 h mean arterial pressure between the 50th and 95th percentile and lower than the 50th percentile, and the blood pressure difference between the groups ranged between 2·7 mm Hg and 3·9 mm Hg in different study years.^1^ Blood pressure separation in our trial (as measured from auscultatory blood pressure) was thus slightly higher than in the ESCAPE study. Control of blood pressure in the standard treatment group (appendix p 10) would have tended to reduce the power of the study to detect a difference in remodelling between groups.

The degree to which cardiac remodelling in children with chronic kidney disease is driven by blood pressure or by other chronic kidney disease-specific factors, including anaemia and increased concentrations of phosphate and parathyroid hormone, has been debated.^7^, ^8^, ^9^, ^10^, ^11^, ^20^ That there was greater reduction of relative wall thickness in the intensive compared with standard treatment group, despite the modest difference in blood pressure achieved, suggests that blood pressure target, or the antihypertensive drugs required to achieve the blood pressure target, play a major part in cardiac remodelling. It is important to note that we cannot distinguish effects of blood pressure reduction alone from those of the drugs used to lower blood pressure. Some antihypertensive drugs, ACE inhibitors or ARBs in particular, might have a blood pressure-independent effect on reverse remodelling and might have played a part in the reduction of relative wall thickness seen in this trial. Our results are generalisable only to regimens that include the use of ACE inhibitors or ARBs as first-line agents (as is usually the case in children with chronic kidney disease).

In the children with left ventricular hypertrophy or abnormal geometry, this regressed in both groups. This finding suggests that there is no benefit in targeting a systolic blood pressure to lower than the 40th percentile. However, several factors point to a blood pressure close to the 50th percentile rather than the 75th percentile as being beneficial. Blood pressure in the standard treatment group was much closer to the lower rather than higher end of the 50th–75th percentile target. This combined with the finding of a blood pressure dependence of cardiac remodelling even with a narrow range suggests that the reverse remodelling seen in the standard treatment group would have been attenuated had the majority of children had blood pressure in the upper range of the 50th–75th percentile. Thus, a target of the 50th percentile would seem close to the optimal for preventing cardiac remodelling. In practical terms, any definition of a target requires an upper limit above which treatment is up-titrated. In our study, there was no adverse effect on safety or renal outcomes of targeting to lower than the 40th percentile. Therefore, until data from larger-scale randomised controlled trials of office blood pressure are available, we suggest targeting to an office systolic blood pressure lower than the 50th percentile with up-titration of treatment at the 50th percentile.

Our trial focused on the use of office auscultatory blood pressure measurement rather than ambulatory blood pressure measurement. Auscultatory blood pressure was chosen because we have previously found it to be a sensitive predictor of left ventricular hypertrophy.^7^ Although ambulatory blood pressure monitoring is recommended in guidelines, many specialist centres in the UK find this too resource intensive for serial monitoring, and some children find serial ambulatory blood pressure monitoring too demanding. Further work to define the best methodology for serial monitoring is required, including a comparison of office auscultatory, automated office, and home blood pressure monitoring with ambulatory blood pressure monitoring. However, the finding in our study that modest differences in standardised office auscultatory blood pressure are associated with reverse remodelling supports the sensitivity of this measurement. Because this study was small and open-label, knowledge of which group the patients were in might have affected the participants' compliance with medications, stress levels at clinic, and perception of side-effects. The majority of our children were followed up for no more than 3 years, and our results cannot necessarily be generalised to long-term outcomes. We have also not assessed the potential cost effectiveness of a more intensive treatment strategy. We did not adjust for multiple testing. However, for the secondary outcome of relative wall thickness, the p value was such that applying a Bonferroni correction for multiple testing of more than five outcomes would still result in a significant difference (p=0·01) in this outcome between intensive versus standard blood pressure reduction.

In conclusion, within the constraints of a small open-label trial, the HOT-KID trial suggests that cardiac remodelling in children with chronic kidney disease is related to blood pressure control and that a target office systolic blood pressure at the 50th percentile is close to the optimal target for preventing increased left ventricular mass.

## Data sharing

Trial-related data are available on reasonable request to the principal investigators.

## Declaration of interests

We declare no competing interests.
